# Complete Genome Sequences of Three *Neisseria gonorrhoeae* Laboratory Reference Strains, Determined Using PacBio Single-Molecule Real-Time Technology

**DOI:** 10.1128/genomeA.01052-15

**Published:** 2015-09-10

**Authors:** A. Jeanine Abrams, David L. Trees, Robert A. Nicholas

**Affiliations:** a Division of STD Prevention, National Center for HIV/AIDS, Viral Hepatitis, STD, and TB Prevention, Centers for Disease Control and Prevention, U.S. Department of Health and Human Services, Atlanta, Georgia, USA; b Departments of Pharmacology and Microbiology and Immunology, University of North Carolina at Carolina at Chapel Hill, Chapel Hill, North Carolina, USA

## Abstract

*Neisseria gonorrhoeae*, the etiological agent that causes the sexually transmitted infection gonorrhea, is a significant public health concern due to the emergence of antimicrobial resistance. We report the complete genome sequences of three reference isolates with varied antimicrobial susceptibility that will aid in elucidating the genetic mechanisms that confer resistance.

## GENOME ANNOUNCEMENT

*Neisseria gonorrhoeae* is a Gram-negative, betaproteobacterium that is the etiological agent responsible for the sexually transmitted infection gonorrhea. In 2008, the World Health Organization estimated 106 million new gonorrhea cases worldwide (1), and the Centers for Disease Control and Prevention (CDC) reported over 300,000 cases in 2012, making it the second most common notifiable infection in the United States (2). The emergence of gonococcal resistance to former first-line antibiotics (e.g., penicillins, tetracyclines, fluoroquinolones, and cephalosporins) has been facilitated by both plasmid- and chromosome-mediated mechanisms (3). In response to the recent emergence of decreased susceptibility to third-generation cephalosporins, the CDC currently recommends the dual-use of ceftriaxone with either azithromycin or doxycycline as treatment for uncomplicated gonorrhea (4).

To better understand the mechanisms associated with antibiotic resistance, we sequenced the complete genomes of three laboratory reference strains with varying degrees of antibiotic susceptibility. Gonococcal strain FA19 is a wild-type strain that was isolated in 1962 from a patient with a disseminated gonococcal infection, and is highly susceptible to penicillin, tetracycline, erythromycin, chloramphenicol, and rifampin (5) (https://www.broadinstitute.org/annotation/genome/neisseria_gonorrhoeae/GenomeDescriptions.html#Strain_fa19). Strain FA6140 was isolated in 1983 from an outbreak of penicillin-resistant gonorrhea in Durham, NC, and it exhibits chromosomally mediated resistance to penicillin, tetracycline, and erythromycin (6). Lastly, strain 35/02 was isolated in 2002 in Sweden, and it exhibits chromosomally mediated resistance to penicillin and reduced susceptibility to the extended-spectrum cephalosporins, cefixime and ceftriaxone (7). Moreover, 35/02 also contains a mosaic *penA* allele with 58 mutations relative to the *penA* gene from FA19 (7); mosaic *penA* alleles are strongly associated with reduced cefixime and ceftriaxone susceptibility in clinical samples (8).

Whole-genome sequencing was conducted using the PacBio RSII platform (Pacific Biosciences, Menlo Park, CA) with P5-C3 chemistry. A single single-molecule real-time (SMRT) cell was used to sequence FA19 (mean coverage of 119-fold), while FA6140 and 35/02 were each sequenced using two SMRT cells (mean coverage of 309-fold and 262-fold, respectively). *De novo* assembly of the genomes was conducted using the hierarchical genome assembly process (HGAP3, SMRTAnalysis 2.3.0) workflow, which included consensus polishing using Quiver (9). Single, closed contigs were assembled for each sequence, and the following genome length and G+C content data were obtained: FA19 (2,232,367 bp, 52.4% G+C content), FA6140 (2,168,698 bp, 52.6% G+C content), and 35/02 (2,173,235 bp, 52.6% G+C content). The genomes were annotated using Prokka version 1.11 (10), and a total of 2,289, 2,230, and 2,228 coding sequences were annotated in FA19, FA6140, and 35/02, respectively. A comparative analysis of these genomes will be reported in a future publication.

### Nucleotide sequence accession numbers.

The complete genome sequences for *N. gonorrhoeae* strains FA19, FA6140, and 35/02 have been deposited in GenBank under the accession numbers CP012026, CP012027, and CP012028, respectively.
